# NiftyPET: a High-throughput Software Platform for High Quantitative Accuracy and Precision PET Imaging and Analysis

**DOI:** 10.1007/s12021-017-9352-y

**Published:** 2017-12-26

**Authors:** Pawel J. Markiewicz, Matthias J. Ehrhardt, Kjell Erlandsson, Philip J. Noonan, Anna Barnes, Jonathan M. Schott, David Atkinson, Simon R. Arridge, Brian F. Hutton, Sebastien Ourselin

**Affiliations:** 1 Translational Imaging Group, CMIC, Department of Medical Physics, Biomedical Engineering, University College London, London, UK; 2 Department for Applied Mathematics and Theoretical Physics, University of Cambridge, Cambridge, UK; 3 Institute of Nuclear Medicine, University College London, London, UK; 4 Dementia Research Centre, University College London, London, UK; 5 Centre for Medical Imaging, University College London, London, UK; 6 Centre for Medical Image Computing (CMIC), University College London, London, UK

**Keywords:** PET, Quantification, Image reconstruction, Uncertainty, Bootstrap, Scatter correction, Random events estimation, Partial volume correction, Normalisation

## Abstract

We present a standalone, scalable and high-throughput software platform for PET image reconstruction and analysis. We focus on high fidelity modelling of the acquisition processes to provide high accuracy and precision quantitative imaging, especially for large axial field of view scanners. All the core routines are implemented using parallel computing available from within the Python package *NiftyPET*, enabling easy access, manipulation and visualisation of data at any processing stage. The pipeline of the platform starts from MR and raw PET input data and is divided into the following processing stages: (1) list-mode data processing; (2) accurate attenuation coefficient map generation; (3) detector normalisation; (4) exact forward and back projection between sinogram and image space; (5) estimation of reduced-variance random events; (6) high accuracy fully 3D estimation of scatter events; (7) voxel-based partial volume correction; (8) region- and voxel-level image analysis. We demonstrate the advantages of this platform using an amyloid brain scan where all the processing is executed from a single and uniform computational environment in Python. The high accuracy acquisition modelling is achieved through span-1 (no axial compression) ray tracing for true, random and scatter events. Furthermore, the platform offers uncertainty estimation of any image derived statistic to facilitate robust tracking of subtle physiological changes in longitudinal studies. The platform also supports the development of new reconstruction and analysis algorithms through restricting the axial field of view to any set of rings covering a region of interest and thus performing fully 3D reconstruction and corrections using real data significantly faster. All the software is available as open source with the accompanying wiki-page and test data.

## Introduction

One of the key aspects of positron emission tomography (PET) is its quantitative capability which allows measurements to be represented in absolute units of radiotracer concentration (e.g., kBq per mL of tissue). Such quantitative measurements have proven to have a significant impact on assessing the response to treatment of many pathologies, such as cancer (Doot et al. 2014) or neurodegenerative disease (Camus et al. 2012). Furthermore, good PET quantitative accuracy and precision are crucial in clinical trials of new therapies (Kinahan et al. 2015; Meikle and Badawi 2005).

However, achieving high quantitative accuracy is dependent on all data correction being performed to the highest possible standard. The correction for photon attenuation has a major impact on quantification, which is not easy to perform, especially in the case of PET/MR scanners where the direct measurement of electron density is not available (electrons are the main cause of photon attenuation and scattering for the photon energy in PET). Other factors, which can significantly undermine quantitative accuracy are detector dead time, variable detector efficiencies, scatter and random coincidence events as well as limited image resolution, which fails to accurately resolve small tissue regions (Meikle and Badawi 2005).

In the following sections, we will comprehensively describe all these factors beginning from the data acquisition through to image reconstruction and analysis, providing advanced computational models and software solutions together with their validation for obtaining high quantitative accuracy and precision.

A number of publicly available software packages have already been proposed, offering a wide choice of reconstruction algorithms. For example, ASPIRE, which is a set of ANSI C routines developed at the University of Michigan for image reconstruction in emission and transmission tomography as well as magnetic resonance imaging (MRI) (Fessler 2013). Another example is NiftyRec, which provides a number of reconstruction algorithms with GPU-accelerated routines for various modalities of emission and transmission computed tomography (Pedemonte et al. 2010). Another important package is the software for tomographic image reconstruction (STIR), which is written in C++ and provides a rich open source library of routines for static and dynamic imaging coupled with scatter correction (Thielemans et al. 2012).

In contrast and in a complimentary manner to the already available software packages, the proposed software platform in the current stage of development puts greater emphasis on high quantitative accuracy and precision obtained through detailed modelling of PET acquisition physics. This is delivered through advanced and computationally expensive models for data correction using high-throughput parallel computing on graphical processing units (GPU). This parallel implementation allows efficient generation of bootstrap replicates of the list-mode data followed by multiple image reconstructions for uncertainty (precision) estimation of any image statistic. The estimation of precision of any image biomarker has become an important factor in the quantitative accuracy of PET, especially in the case of clinical trials and longitudinal imaging in neurodegeneration and cancer (Kinahan et al. 2015). For example, it has been shown that the changes of amyloid deposition over time are very subtle and often within the test/retest variability of PET (Landau et al. 2015). Therefore, the provided knowledge of uncertainty of any image statistic can be of significant value in preventing false positive findings.

Based on the high accuracy quantitative reconstruction, the platform is easily extended to image post-processing, which we demonstrate as an example application on amyloid brain imaging. Similar quantification software is available from Siemens, called “*syngo* ^®;^.PET Amyloid Plaque”, or the CortexID Suite by GE Healthcare, which both facilitate quantification of amyloid plaque deposits in the brain (Siemens; Peyrat et al. 2012). The image analysis we propose here differs in that it delivers the highest possible quantitative accuracy with precision (uncertainty distributions) of any regional/voxel value in the native PET image space (as opposed to the MNI space used in Peyrat et al. (2012)). It estimates the precision through multiple list-mode bootstrap replicates, for each of which the whole process of quantifying amyloid is repeated many times (Markiewicz et al. 2016a). Every processing stage of this pipeline is fully controlled from within Python, allowing for quality control and validation of all PET data corrections as well as fine tuning for any given imaging task. In addition, the acquisition model can be limited to an arbitrary number of detector rings, thus while still supporting real measurements, it allows for extremely fast data processing which is useful for the discovery of new computational algorithms and quantitative imaging methods (Ehrhardt et al. 2016).

In the following sections we expand on all the stages of data processing for accurate PET acquisition modelling, image reconstruction and analysis within the proposed uniform computational Python environment. We start with the acquired raw data and end with accurate estimates of amyloid deposition in the brain accompanied with the estimated precision of the deposition.

## Methods: Stages of Quantitative Data Processing

**Fig. 1:**
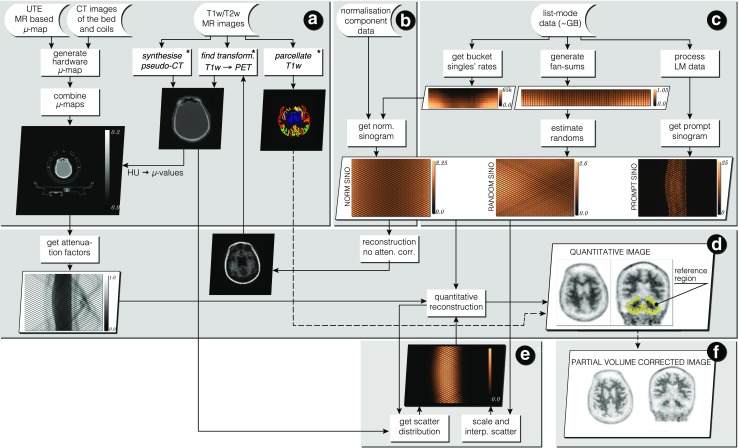
Infrastructure for standalone PET image reconstruction and analysis of PET/MR brain data using amyloid PET tracer. Section *A* presents the image input data with necessary processing for generating accurate hardware and object *μ*-maps as well as parcellation of the brain image into standard anatomical regions (used in reconstruction and analysis sections *D* and *F*). In section *B* the normalisation component data is used to generate single factors for each sinogram bin, with the use of bucket singles—the output from list-mode (LM) processing in section *C*. Apart from singles’ buckets, the LM processing in section *C* generates prompt and delayeds sinograms, and fan sums, which are used for estimating low noise randoms in each sinogram bin. In stage *D* image reconstruction and analysis takes place with a heavy use of forward and back projectors. Note that the attenuation factors are generated with the forward projector. Section *E* contains scatter estimation which is coupled with image reconstruction—the scatter is updated every time a better image estimation of the radiotracer distribution is available. Using the parcellation from *A* and the system’s point spread function (PSF), the reconstructed image is corrected for the partial volume effect in section *F*. ^∗^ *External software packages*

All the processing stages are presented within the complete infrastructure depicted in Fig. 1 using an amyloid brain scan acquired on the Siemens Biograph mMR. The participant was taking part in “Insight 46”—a neuroscience sub-study of the Medical Research Council National Survey of Health and Development (Lane et al. 2017). The input data include the attenuation coefficient maps (*μ*-maps) of the hardware and subject (stage *A*), normalisation component data (stage *B*) and the list-mode data (stage *C*). Optionally, T1 and/or T2 weighted MR images are provided for brain parcellation (Cardoso et al. 2015) used in partial volume correction (PVC) and regional analysis as well as for generating a more accurate subject *μ*-map (Burgos et al. 2015). In this work, we put a greater emphasis on the quantitative image reconstruction and analysis in: forward and back projectors for image reconstruction (stage *D*); fully 3D estimation of scatter events (stage *E*); and voxel-wise partial volume correction using MRI brain parcellations (stage *F*).

### List-mode Data Processing

The list-mode data are rich in spatio-temporal information, which typically require a large amount of memory, in the order of GB, for a clinical study. Since it is challenging to process such an amount of data in a fast and efficient way, we have devised a novel method using the GPU for rapid processing of list-mode datasets (Fig. 1c), details of which are covered in our previous publication (Markiewicz et al. 2016a). Here we will present a concise outline of this method with some additional details.

**Fig. 2:**
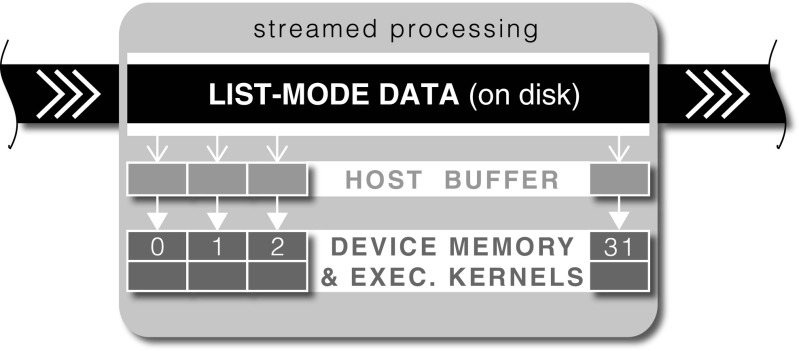
Workflow of concurrent list-mode (LM) processing. The LM data is divided into data chunks and processed by 32 CUDA streams at any given time. On processing completion by any stream, a new LM data chunk is read from the disk and processed asynchronously until all LM data is read and processed

The workflow of the list-mode (LM) data processing is depicted in Fig. 2. The key aspect of fast LM processing is the concurrent execution of device kernels (GPU functions) using 32 CUDA streams (Harris 2012a), while copying the next chunks of LM data in advance from disk to the host (CPU) buffer and then to the device (GPU) memory. The overlapped data transfer and execution allows the exploitation of data transfer lag for GPU processing (Harris 2012b). In our current implementation the buffer size is around 1.6 GB and divided into 32 data chunks of 50 MB each, processed by the corresponding 32 CUDA streams.

The output of the LM processing includes the following: $\blacktriangleright $ *Prompt and delayed sinograms for static or dynamic acquisitions* in (i) span-1 with no axial compression resulting in 4084 sinograms for the Biography mMR scanner; (ii) span-11 with axial compression resulting in 837 sinograms; or (iii) using single slice rebinning (SSR) with only 127 sinograms. $\blacktriangleright $ *The head curve*, which is the total counts per second for the prompt and delayed events recorded in the list data. $\blacktriangleright $ *General motion detection* is obtained based on the centre of the radioactivity mass in the axial dimension. $\blacktriangleright $ *Fan sums for delayed events.* The fan sums are used as an input for generating reduced-noise randoms estimates using maximum likelihood with the Poisson model for random events (cf. “Estimation of Random Events”). $\blacktriangleright $ *Bucket singles rates* are reported approximately every other second for each bucket (8 buckets axially × 28 buckets transaxially = 224) and used for detector dead time correction. $\blacktriangleright $ *Sagittal and coronal dynamic projection views* are created every fourth second of acquisition. These views are used to generate videos for visual assessment of the acquisition for quality control. An example video for a case with significant motion is available at https://vimeo.com/129831482.

### Detector Normalisation

The sensitivity of each detector (including crystals and electronics), and thus the sensitivity of each line of response (LOR), varies significantly. This causes considerable quantitative errors and visual artefacts, mostly of high frequency (Meikle and Badawi 2005). Therefore, for high quantitative accuracy imaging these effects have to be corrected and a dedicated normalisation scan is performed to obtain all the necessary normalisation components (factors) reflecting the variable detection sensitivity. The overall normalisation coefficients for each LOR are modelled as a product of transaxial and axial components which include, among others, the geometric effects and intrinsic crystal efficiencies (Casey et al. 1996; Badawi and Marsden 1999). These components are provided by the scanner for each PET acquisition with some of the components being independently calculated within the *NiftyPET* package for imaging without axial compression of the projection data (not supported by the vendor). By combining these components with the single rates from LM data, full normalisation factor sinograms are calculated.

#### Transaxial Normalisation Factors

The transaxial components include: $\blacktriangleright $ *Geometric effects* factors, which account for the efficiency differences associated with the distance of the LOR from the transaxial iso-centre of the scanner. Note that these factors apply only to the true events and not scatter events. $\blacktriangleright $ *Crystal interference:* These capture the varying detection efficiency due to the relative position of one crystal within a block of detectors. $\blacktriangleright $ *Detector efficiencies:* These describe the random variations in crystal efficiencies due to the slightly varying crystal quality, as well as different photodetector gains when converting the weak crystal light output into a corresponding electrical signal. $\blacktriangleright $ *Detector dead time:* These characterise the drop of efficiency at higher count rates. The drop is caused by the minimum amount of time, which is required to elapse between two events in order for them to be recorded as two distinct events. For high count rates it is more probable that the events will not be separated and likely to be rejected altogether. The dead-time is modelled by two components (Meikle and Badawi 2005; Evans 1955): the *paralysable* and *non-paralysable* components modulated by the detector single rates, which are measured at the buckets level to capture the spatially variant dead-time effect (see “List-mode Data Processing” and Markiewicz et al. 2016a).

#### Axial Normalisation Factors

##### Axial effects for true events

**Fig. 3:**
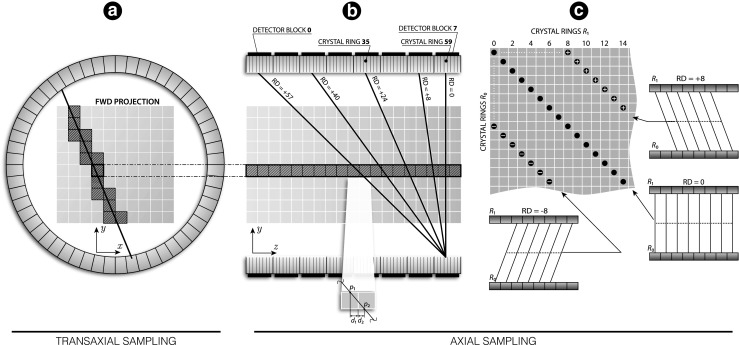
**Forward projection model used in forward and back projection:** Ray-driven calculations are decomposed into transaxial (*A*) and axial (*B*) components. For a chosen transaxial voxel position, all the computations are performed axially, leading to storing the projection data along the Michelogram diagonals (*C*) (shown is Michelogram patch with sampling along the direct [*R* *D* = 0] and oblique [*R* *D* = ± 8] sinograms)

Axial factors capture the varying efficiency of each direct or oblique sinogram due to the axial block profile, with the assumption that the transaxial block profile (crystal interference above) is accounted for. For the Biograph mMR, the component is provided as an array of 837 factors for each axially compressed sinogram in span-11. Each compressed sinogram can consist of up to 6 uncompressed (span-1) sinograms. Axial sampling in span-1 and span-11 can well be represented by the Michelogram (Bailey 2005) (see Fig. 3c; cf. “Results and Discussion”, Fig. 9a). Since, by default, the scanner does not output span-1 axial factors, these are derived here from a very high statistics acquisition of the ^68^Ge cylindrical phantom, scanned for 24 hours, and from the provided axial factors for span-11. The contribution of span-1 axial factors to the given span-11 axial factors, *𝜖* *u* *v*(11), is ‘decoded’ according to the following formula:

1 $$ \epsilon_{uv}^{(1)} = N_{uv} \epsilon_{uv}^{(11)} P_{uv}^{(1)} / P_{uv}^{(11)}, $$

where $P_{uv}^{(1)}$ is the span-1 Michelogram of the emission phantom prompt data between rings *u* and *v* (cf. “Results and Discussion”, Fig. 9b; note the varying efficiencies across the detector blocks [8x8 crystals]), $P_{uv}^{(11)}$ is its span-11 equivalent and *N*_*uv*_ is the number of sinograms contributing to the span-11 group containing rings *u* and *v*.

##### Customisable axial FOV

The above extension from span-11 to span-1 normalisation made possible the customisation of the axial FOV (64 rings) into smaller setups of detector rings. This enables significantly faster reconstruction times, which is particularly useful for developing new reconstruction and analysis methods when a large number of tests have to be carried out.

##### Axial effects for scatter events

The presented software includes a novel voxel-based single scatter model (see “Fully 3D Scatter Model”), which requires specific axial normalisation factors to account for the scatter specific block effects. The normalisation is performed in the span-1 or span-11 sinogram space, while the scatter scaling to the prompt data is performed using single slice rebinned (SSR) data for higher counting statistics. The normalisation factors are found as ratios between the SSR data and the corresponding span-1/span-11 sinogram data, using the long 24 hour phantom acquisition to ensure good statistics.

### Generation of the *μ*-map (Specific to PET/MR Scanners)

The accurate spatial distribution of the linear attenuation coefficient (also known as the *μ*-map in units of cm^− 1^) is crucial for quantitative PET imaging. Since MR cannot measure electron density accurately (like the CT scans in PET/CT scanners or transmission scans in older PET scanners), there are specific workarounds for generating hardware and subject *μ*-maps for use in PET/MR scanners.

#### The hardware *μ*-map

Since the bed cannot be imaged with MR, high resolution CT images of the patient bed (table) and the MR coils are supplied with the scanner. The images of different hardware components can be found in the scanner’s file system with hardware components such as the head and neck coil, the spine coil and the patient bed. The specific part of the hardware *μ*-map for any given image study has to be separately generated based on additional information about the table position relative to the iso-centre of the scanner. Depending on the imaging settings, only some parts of the hardware are in the field of view and only those parts have to be included in the *μ*-map by appropriate image resampling of the high resolution CT-based images into the PET space.

#### The patient *μ*-map

In addition to the hardware *μ*-map, information about the object’s electron density is required for attenuation correction. For brain imaging, the software offers a choice between the *μ*-map provided by the scanner or the *μ*-map generated using the pseudo-CT (pCT) synthesis from T1 and T2 weighted (T1w/T2w) MR images (Burgos et al. 2015). The multi-atlas CT synthesis method provides a significant improvement in PET quantitative accuracy when compared to the ultra-short echo time (UTE)-based attenuation correction (as provided by the vendor). In this method, atlases of multiple pairs of aligned MR and CT images from different subjects are used to generate a synthetic CT image, called also a pCT image in the original, target MR space. It is possible to generate pseudo-CT images using a web application: http://cmictig.cs.ucl.ac.uk/niftyweb/program.php?p=PCT with the output image in NIfTI format, in the T1w/T2w image space. The CT values, which are expressed in HU, are converted to linear attenuation coefficients using a piecewise linear transformation (Burger et al. 2002).

Since it is likely that patient motion will occur between the T1w/T2w and PET acquisitions, the software allows creation of a reference PET image (an average image or any dynamic time frame), which is reconstructed without attenuation correction, and to which the MR images are then registered (Modat et al. 2014). The resulting transformation is used to resample the pCT image into the correct position and resolution of the PET image of the time frame.

### Forward Model for Iterative Image Reconstruction

The quantitative information about the spatial distribution of radioactivity is carried by photons travelling along straight paths between two detectors without interacting with the matter of the patient body or the scanner hardware (e.g., the table and coils). In this work the continuous radioactivity distribution *f* is discretised and approximated by a set of *J* voxels, i.e., $f(\mathbf {x}) = {\sum }_{j = 1}^{J}n_{j}v_{j}(\mathbf {x})$, with *n*_*j*_ being the radioactivity concentration within the finite volume of voxel *j* defined by a 3D top-hat function *v*_*j*_(**x**) (Leahy and Qi 2000). The underlying radioactivity distribution is commonly estimated using iterative methods, which have the key advantage of the ability to include more sophisticated models of the PET acquisition process (Alessio et al. 2006), based on the discrete formulation:

2 $$ q_{i} = \sum\limits_{j = 1}^{J} p_{ij}n_{j}, $$

where *q*_*i*_ is the expected data in the *i*-th LOR from the distribution {*n*_*j*_}_*j*= 1,…,*J*_, and *p*_*ij*_ is an element of the system matrix **P** representing the probability of positron emission in voxel *j* resulting in detection of an event by *i*-th LOR. These methods require computationally costly forward and back projections from the image to projection space and vice versa, using integrals along the LORs. Such calculations are especially costly for large axial field of view (FOV) scanners, like the Biograph mMR scanner. However, since there are many forward and back projections for which the integral calculations are independent, parallel computing architectures based on graphics processing units (GPUs) can be successfully employed allowing very fast implementations (Markiewicz et al. 2014; Ha et al. 2013).

The very large number of LORs and voxels, especially in the large axial field of view scanners, prohibits storing the system matrix in the computer memory for subsequent reuse. Therefore, the coefficients of the system matrix are calculated on the fly using the ray-driven Siddon algorithm (Jacobs et al. 1998; Siddon 1985). The algorithm allows for exact calculations of the intersection length of photon trajectories passing through any voxel along the path between both detectors of an LOR (Fig. 3a and b).

For the large number of detector rings (64 in the Biograph mMR scanner), the ray tracing was decomposed into axial and transaxial components. The transaxial component consists of voxel intersections on the *x* − *y* image plane, which are the same for any axial voxel row through which photon rays are traced (Fig. 3b). The transaxial component is pre-computed first and then stored in memory to be then used for actual 3D ray-tracing by projecting the transaxial intersections onto all possible *z* directions defined by an allowed ring difference (*R* *D* = *r*_1_ − *r*_0_, *R* *D* ≤ 60). Note, that all ray tracing is calculated independently for each detector pair, and thus, for span-11 the ray tracing is always performed in span-1, followed by ray compression to form span-11 sinograms.

Although, the Biograph mMR scanner is used here, *NiftyPET* allows other cylindrical geometries to be added through a simple parametrisation of scanner geometry decomposed into the transaxial and axial parts. For the axial part, it requires the number of detector rings and their size, while for the transaxial part, it requires the ring diameter, number of detector blocks and their size as well as the number of crystals per block and their size.

In this work, images were reconstructed using the ordered subsets expectation maximisation (OS EM) algorithm (Hudson and Larkin 1994). For the Biograph mMR, *N* = 14 balanced subsets were used, obtained by dividing the projection data along sinogram angles (252) into *N* subsets. Therefore, each subset consisted of 18 sinogram angles × 344 radial bins × 4084 direct/oblique sinograms. The correction for randoms (“Estimation of Random Events”) and scatter (“Fully 3D Scatter Model”) was performed using two additive terms to the forward model of Eq. (2) in the reconstruction procedure (Tamal et al. 2006). The scatter estimate is updated at each iteration of image reconstruction.

#### GPU Implementation

To achieve high throughput processing, the projection and image data are reorganised in the device memory allowing high bandwidth (coalesced) memory access. The image and projection data are rearranged such that the fastest changing index for the image and projection data is along axial direction. Therefore, this leads to axially-driven calculations, which allow more efficient use of the L2 cache. Consider an axial image row (127 voxels for the Biograph mMR) of fixed transaxial position as shown in Fig. 3b and a pair of transaxial detectors forming a set of possible LORs intersecting the image row. The axial intersections for these voxels are calculated for all oblique and direct sinograms, after combining them with a single pre-calculated transaxial intersection length for one such axial row (cf. Fig. 3a). This process is then repeated for all voxels being intercepted by the chosen LOR, which leads to the projection data being stored consecutively along the diagonals of the Michelogram and thus ensuring coalesced load and store operations (NVIDIA 2017a) (Fig. 3c).

Since the projection paths in the cylindrical scanner geometry vary depending on the radial projection position, the computational load will vary correspondingly for each projection bin (Hong et al. 2007). Therefore, to keep threads well-balanced with similar workload, and thus maintaining high throughput by minimising idle threads, the projection data are first sampled along sinogram angles followed by the radial projection sampling. The reason for this is that the same radial projection position will have similar intersection length through a circular FOV for any sinogram angle, and hence threads with similar calculation load are bundled together and executed in parallel more efficiently.

#### Technical specifications for the Biograph mMR projector

Forward and back projections are executed in three parts: (i) the 64 direct sinograms are executed by a grid of 68516 CUDA blocks, whose number corresponds to the total number of sinogram bins without crystal gaps and each block is comprised of 64 threads (see documentation (NVIDIA 2017a) for details); (ii) the next 1024 oblique sinograms are executed by a grid of 68516 CUDA blocks, each with 1024 threads (maximum number of threads per block for NVIDIA architectures with a compute capability of 3.5); (iii) the remaining oblique sinograms are executed with the same parameters as (ii). Forward projection takes around 3 seconds on the NVIDIA K20 Tesla. The back projection takes 3 seconds more due to the resolving of race hazards using the CUDA atomic operations (race hazards are created by multiple projection bins accessing the same image voxel at the same time). Currently, no symmetries are used for speeding up the calculations as in Hong et al. (2007), but such an approach is under development.

### Estimation of Random Events

#### Measurement of random events

For quantitative imaging, the random coincidences have to be accurately measured for each detector pair. For the Siemens Biograph mMR scanner, the random coincidences are measured using the delayed time window method (Meikle and Badawi 2005) (p.96), in which the true and scatter coincidences are eliminated from such a delayed acquisition, leaving only the estimate of randoms. Since such estimates can be very noisy, especially for short time frames (Hogg et al. 2002) (cf. Fig. 7b in “Results and Discussion”), we implemented a maximum likelihood method for reducing the noise of the random events estimates.

#### GPU-based random events estimation

The rate of measured random events between detectors *i* and *j* within the time coincidence window 2*τ* can be approximated using singles rates *S*_*i*_ and *S*_*j*_:

3 $$ R_{ij} \simeq 2\tau S_{i}S_{j}. $$

**Fig. 4:**
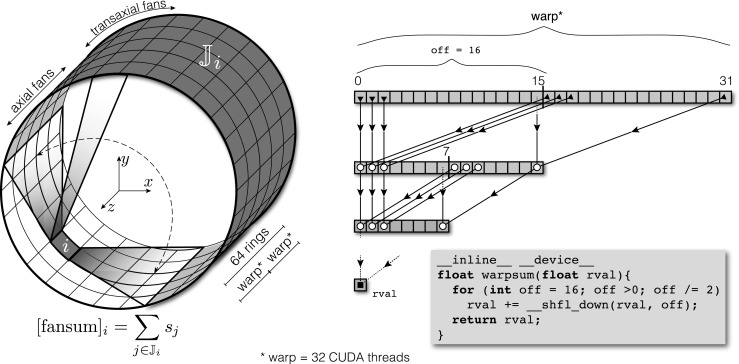
**Calculation of 3D fan sums for each detector** ***i***: The sum is first calculated axially by forming axial fans of 64 rings and then transaxially by forming transaxial fans, such that all detectors $\mathbb {J}_{i}$ in coincidence with detector *i* are summed (three axial fans are shown within the detector rings where two of the fans are on extreme ends). One axial fan sum is calculated by two CUDA warps (see the figure on the right), where each warp consists of 32 CUDA threads executed in parallel. The values stored in 32 registers (one register per thread) are reduced to one sum through fast parallel reductions obtained through rapid communications between the threads and facilitated by CUDA *shuffle instructions* (Luitjens 2014). The same is done for the other warp to form one axial fan sum. Repeating it over all transaxial detectors will constitute a full fan sum for detector *i*

Since the random events follow Poisson statistics, the expected values of the estimated random data are found using the maximum likelihood (ML) approach based on (3) (Panin et al. 2007):

4 $$ S_{i}^{(k + 1)} = \frac{1}{2} S_{i}^{(k)} + \frac{1}{2} \frac{ {\sum}_{j\in \mathbb{J}_{i}} d_{ij} }{ {\sum}_{j\in \mathbb{J}_{i}} 2\tau S_{j}^{(k)} }, $$

where ${\sum }_{j{\in }\mathbb {J}_{i}}d_{ij}$ are the fan sums found while processing the list-mode data for delayed events (for more details see “List-mode Data Processing” and Markiewicz et al. (2016a) and Panin et al. (2007)). The fan sums in the denominator of Eq. 4 are calculated at each iteration on the GPU exploiting inter-thread communication for very fast reductions using CUDA *shuffle instructions* (Luitjens 2014). The 3D fan sums are found first for axial fans as shown in Fig. 4 and then for transaxial fan sums for any given detector *i*. The random event sinograms in span-1 are found by applying (3) to each sinogram bin (span-11 sinograms are found by reducing span-1 sinograms accordingly).

### Fully 3D Scatter Model

Another key component for accurate quantitative imaging is scatter correction. In this work we adopted a fully 3D, voxel-driven scatter model (VSM) which is based on single scatter simulation. The key difference of this model to the established methods of Ollinger (1996) and Watson (2000) and their newer extensions (Iatrou et al. 2006; Kim and Ye 2011), which use a line of response (LOR) driven approach, is that in the proposed voxel-driven approach each emission voxel is treated independently resulting in a separate 3D probability scatter sinogram for each emission voxel. The global scatter response is found by summing all the scatter contributions from each emitting voxel. This feature can prove useful in modelling the scatter component in the system matrix (Tamal et al. 2006; Markiewicz et al. 2007), and enables more accurate TOF scatter estimates (Markiewicz et al. 2016b). Furthermore, it allows greater control over the input emission (its resolution and the radioactivity concentration threshold, over which voxels are considered for scatter estimation).

#### Methods

**Fig. 5:**
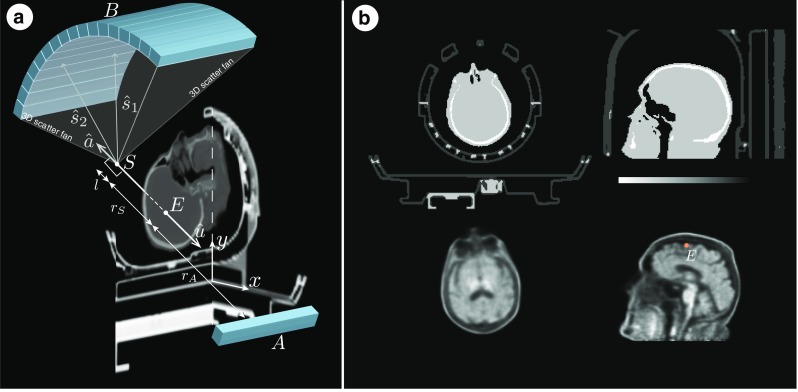
**Scatter modelling and validation setup**. **a:** Voxel-driven scatter model (VSM) based on single scatter simulation. It assumes that photons emitted at *E* (shown in the sagittal plane) along $\hat {\mathbf {u}}$ are unscattered and detected while the opposing photons emitted along $\hat {\mathbf {a}}$ are assumed scattered from the original trajectory and then detected on the detector ring. **b:** Validation Monte Carlo setup using SimSET with ^18^F-florbetapir and the Siemens Biograph mMR geometry. Top row includes the transaxial and sagittal *μ*-map images whereas the bottom row consists of the corresponding emission images for brain and point source (marked point *E*) simulations

Consider a positron emission at *E* giving rise to a photon pair emitted such that one photon is detected unscattered at *A* while the other one is incident on scattering patch *S*. The unscattered photon trajectory ($\hat {\mathbf {a}}=-\hat {\mathbf {u}}$) is defined by detector *A* and emission location *E* (shown in the sagittal plane in Fig. 5a). The probability of photons being incident on *S* is:

5 $$ P_{\mathrm{i}}(SEA) = \varepsilon_{A} \exp\left[-{\int}_{-r_{A}}^{r_{S}} \mu(l \hat{\mathbf{a}} + \mathbf{e})\mathrm{d} l\right], $$

where *ε*_*A*_ is the geometric efficiency of detecting a photon emitted at *E* (specified by position vector **e**), *r*_*A*_ is the distance between *E* and the opposing detector along path $\hat {\mathbf {u}}$ and *r*_*S*_ is the distance between *E* and the beginning of patch *S* along path $\hat {\mathbf {a}}$. Each scatter patch is represented by a single point, **s** = (*s*_*x*_,*s*_*y*_,*s*_*z*_), from which photons are assumed to be scattered. Therefore, the absolute probability that a photon emitted around *E* will scatter at *S* (i.e., along the length *l*_*S*_ of the scattering patch) while the other photon will be received unscattered at crystal *A* is given by

6 $$ P_{\mathrm{s}}(SEA) = P_{\mathrm{i}}(SEA) \left\{ 1 - \exp\left[-{\int}_{-l_{\mathrm{s}}/2}^{l_{s}/2} \mu(l\hat{\mathbf{a}}+\mathbf{s})\mathrm{d} l\right] \right\}. $$

The probability of photons scattering from *S* towards a given detector *B* is found using the Klein-Nishina (K-N) cross-section, d*σ*_e_/dΩ, for unpolarised radiation and the solid angle Ω_*B*_ subtended by detector *B* at *S*, leading to the total absolute probability *P*_s_(*B* *S* *E* *A*) that a positron emitted at *E* will result in an unscattered photon detected at *A* and the paired scattered photon at *B*:

7 $$ P_{\mathrm{s}}(BSEA) \,=\, P_{\mathrm{s}}(SEA) \frac{{\Omega}_{B}}{\sigma_{\mathrm{e}}} \left( \frac{\mathrm{d} \sigma_{\mathrm{e}}}{\mathrm{d} {\Omega}_{B}}\right) \exp\left[-c_{B}{\int}_{0}^{r_{B}} \mu(l\hat{\mathbf{s}}_{i} + \mathbf{s})\mathrm{d} l\right], $$

where *σ*_e_ is the total K-N electronic cross-section, *c*_*B*_ is the factor accounting for the changed photon energy after scattering towards *B*. The 3D scatter response to emission point *E* is found by accounting for all detectors receiving unscattered photons. This procedure is repeated for all the possible emission voxels to estimate the full scatter sinogram.

#### Implementation

In this model, a sub-sample *K*_S_ of all available detectors *K* are considered to receive the unscattered photons and their paired scattered photons, all emitted at the vicinity of any point *E*. The model requires as an input the current estimate of radioactivity distribution and the *μ*-map images. Due to the efficient and high-throughput computational model, both images can be represented in high resolution, allowing high accuracy and precision. For ^18^F-florbetapir radiotracer, the *μ*-map was down-scaled by a factor of two (from [127 × 344 × 344] to [63 × 172 × 172] using 4 mm^3^ voxels) to allow high accuracy of photon attenuation calculations. The radioactivity image was down-scaled independently from the *μ*-map by a factor of 3, resulting in [43 × 114 × 114] images. The independent scaling allows for greater control of the trade-off between accuracy and computational time.

One of the advantages of this voxel-driven approach is its suitability for multi-level parallel GPU computing (or using other parallel computing architectures), with all the emission voxels being separated and calculated independently in the top level of parallelism. Then, in the lower level of parallelism, all the detectors receiving unscattered photons are considered separately: first axially with 8 detector rings out of all 64 rings (resulting in 1:8 axial sampling) and then transaxially with 64 detectors out of 448 (resulting in 1:7 transaxial sampling, similar to the axial sampling using the Siemens Biograph mMR geometry). The next (lower) level of parallelism is used for calculating the paths of scattered photons detected by 8 axial rings and 32 transaxial detectors, which form a 3D fan of the *basic scatter distribution* originating at scattering point *S* on trajectory $\hat {\mathbf {a}}$ and ending on the detector ring (Fig. 5). The lowest level of parallelism is employed on each CUDA warp (a group of 32 CUDA threads scheduled and executed simultaneously) with fast reductions using *shuffle instructions* introduced in the NVIDIA’s Kepler architecture, facilitating rapid tracing of photon rays through the attenuating medium (NVIDIA 2012).

The tracing involves calculating the survival probability of photons arriving at scattering patch *S* or a detector, for both scattered and unscattered photons. The photon tracing along rays from each voxel to any detector is calculated once and stored in a look-up table (LUT) in the device memory using 16-bit integer format with a global floating point scaling factor. Since only a subset of axial and transaxial detectors are used in scatter estimation, the full size scatter sinograms are found using intra-sinogram bi-cubic interpolation for individual sinograms and inter-sinogram bi-linear interpolation performed in the Michelogram space. Dedicated scatter normalisation efficiencies are used for each individual oblique sinogram (see “??”).

The last step is to scale the scatter sinogram to the prompt data to account for multiple scatter and scatter from outside the FOV. Since all the quantitative proportions between scatter sinograms are maintained within the 3D model, the scaling factors are obtained using the weighted least squares method applied to reduced sinograms for high count levels. Finally, this process is followed by scatter specific normalisation in span-1 or span-11 (cf. “Axial Normalisation Factors”).

#### Monte Carlo Validation

The proposed scatter model was validated using Monte Carlo simulation toolkit SimSET (Lewellen et al. 1998) with two setups: (i) a point source within an attenuating medium and (ii) simulated amyloid ^18^F-florbetapir brain scan (Fig. 5b). In both cases the geometry of the Siemens Biograph mMR was used. The *μ*-map for both setups is taken from a real amyloid brain scan, including the patient’s head and neck, the table and the head coil. The location of the simulated point source is marked with point *E* in Fig. 5b, whereas the whole brain simulated radioactivity is taken from a real reconstructed ^18^F-florbetapir brain scan. In the case of the point source, a total of 2 × 10^10^ events were simulated. For the brain scan, a total of 3 × 10^10^ events were simulated across the whole brain.

### Partial Volume Correction

#### Methods

Partial volume correction (PVC) is applied in order to improve both the qualitative and quantitative aspects of the reconstructed PET images by correcting for the degrading effects of the limited spatial resolution. While many PVC methods have been proposed (see e.g., Erlandsson et al. 2012 for review), here we have chosen to implement the “iterative Yang” (iY) method, an iterative version of a method proposed by Yang et al. (1996). This method utilises a segmented anatomical image to correct for the spill-over between different regions on a voxel-by-voxel basis, as modelled by the PSF of the scanner. There is no correction for blurring between voxels within the same region, however. For these reasons the method does not suffer from the excessive noise-amplification and ringing artefacts associated with standard de-convolution algorithms, and produces results similar to those of the RBV method (Thomas et al. 2011). The iY method can be described as follows

8 $$ \hat{f}^{(k)}(\mathbf{x}) = g(\mathbf{x})\frac{b^{(k-1)}(\mathbf{x})} {h(\mathbf{x}) \ast b^{(k-1)}(\mathbf{x})} $$

with

9 $$ b^{(k)}(\mathbf{x}) = \sum\limits_{i = 1}^{N} a^{(k)}_{i} I_{i}(\mathbf{x}) $$

and

10 $$ a^{(k)}_{i} = \frac{\int I_{i}(\mathbf{x}) \hat{f}^{(k)}(\mathbf{x}) d\mathbf{x} } {\int I_{i}(\mathbf{x}) d\mathbf{x}}; i = 1,...,N, $$

where $\hat {f}^{(k)}(\mathbf {x})$ is the corrected image after *k* iterations, *g*(**x**) is the original image, *h*(**x**) is the PSF of the system, *I*_*i*_(**x**) is the indicator function for region *i*, *N* is the number of regions (which is unlimited), **x** is a 3D spatial coordinate, ∗ represents the convolution operator, and the integral is evaluated over the entire FOV. The procedure is initialised with: $\hat {f}^{(0)}(\mathbf {x}) = g(\mathbf {x})$ and typically converges after approximately 10 iterations.

#### Implementation

**Fig. 6:**
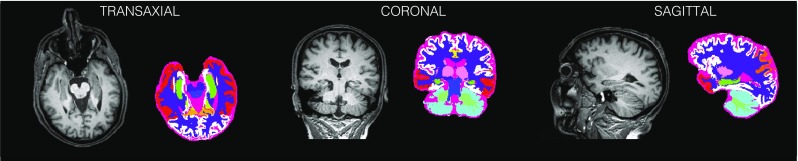
Brain parcellation based on T1w MR image for partial volume correction

In practice, PVC is performed in the MRI domain with binary parcellations (Fig. 6), so the PET image first needs to be up-sampled, as the voxel-size is typically smaller in MRI than in PET. The parcellation is obtained using a multi-atlas segmentation propagation strategy (Cardoso et al. 2015) based on a T1w MR image which was parcellated into 144 regions of interest (ROI), which are then grouped into relevant ROIs for amyloid imaging, including: the cerebellar white and great matter, pons, brain stem, cingulate gyrus, hippocampus, precuneus, parietal and temporal lobes and the whole neocortex. The previously obtained global transformations (from the T1w MR to the PET space, see “Generation of the *μ*-map (Specific to PET/MR Scanners)”) were then used to propagate the regions of interest from the MRI space to the PET space.

The most computationally expensive operation in this PVC algorithm is the 3D convolution, which is expensive for the higher resolution input images (the PET image is upsampled to a resolution of at least that of the T1w MR). The convolution was implemented on the GPU using the method of separable kernels (see the NVIDIA’s CUDA SDK algorithm of 2D separable filters, which we extended to 3D PET images (Podlozhnyuk 2007)). The 3D kernel is decomposed into three one-dimensional filters: one for the transaxial image rows, one for the transaxial columns and one for the axial rows. Therefore, a separable kernel convolution can be divided into three consecutive one-dimensional convolution operations. It requires only *U* + *V* + *W* multiplications for each output voxel as opposed to the standard convolution requiring *U* ∗ *V* ∗ *W* multiplications (*U*,*V*,*W* are the sizes of the kernel in *x*,*y*,*z* directions, respectively). The kernel itself is based on point source measurements on the Biograph mMR scanner, followed by parametrisation of the measured kernel shape through fitting two Gaussians for each one-dimensional kernel.

## Results and Discussion

To demonstrate and validate the quantitative accuracy of *NiftyPET* package as a whole, we used two list-mode datasets acquired on the Biograph mMR: (i) a 24-hour acquisition of a ^68^Ge cylindrical phantom, with a diameter of 20cm; and (ii) a brain scan in an amyloid negative participant using the ^18^F-florbetapir radiotracer. The long acquisition phantom data is useful for testing all the quantitative corrections, particularly scatter (when done correctly it will result in uniform images) and normalisation (any high frequencies in the reconstructed image would suggest normalisation inaccuracies). Using the brain scan dataset, we will demonstrate the whole chain of brain imaging and analysis, including: (i) quantitative data modelling, (ii) image reconstruction followed by (iii) partial volume correction and (iv) voxel/regional-level image analysis for amyloid brain deposition. Furthermore, the capabilities of the *NiftyPET* package go beyond the usual point-estimate image reconstruction offering estimates of the distributions of regional and voxel value uncertainties through the use of list-mode bootstrapping. All the presented results here will be shared through Jupyter Notebooks—an excellent and free tool for sharing, communicating, and most of all, replicating data analysis.

### List-mode data processing output

**Fig. 7:**
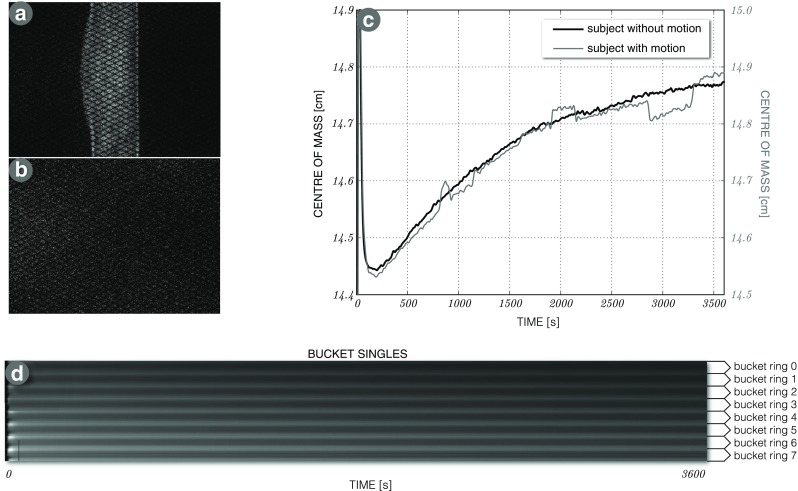
**Selected output of list-mode processing**: **(a, b)** prompt and delayed sinograms for a 10 minute ^18^F-florbetapir acquisition. **c** Fast motion detection based on the centre of mass of axial radioactivity distribution—shown for subject with motion (grey curve) and without motion (black curve) over the 60 minute acquisition. **d** Dynamic singles rates reported as 224 buckets for the whole acquisition over time

Example output is shown for an amyloid (^18^F-florbetapir) brain scan in Fig. 7 (for a comprehensive description see Markiewicz et al. (2016a)). Figure 7a and b show the prompt and delayed event sinograms for the last 10 minute time frame of the total 60 minute acquisition. Figure 7c shows two radioactivity centre of mass curves for a subject with minimal motion (black curve) and a subject with significant motion (grey). It can be noted that motion patterns are distinct from the patterns of slowly changing tracer kinetics. Figure 7d shows the dynamic readout of the singles rates per bucket (image *y*-axis) over acquisition time (image *x*-axis).

### Transaxial normalisation factors

**Fig. 8:**
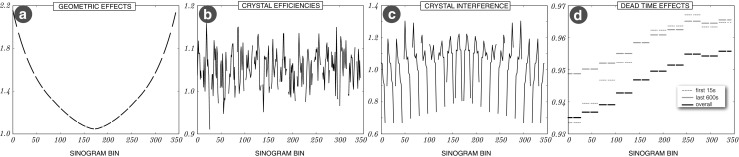
**Transaxial sinogram profiles of normalisation factors for four different components**: **a** in-plane geometric effects; **b** crystal efficiencies—for each sinogram bin the factor is a sum of products of two crystal efficiencies per each LOR contributing to the sinogram bin; **c** crystal interference (transaxial block effects); **d** detector dead time for each detector sinogram bin which can be a combination of multiple LORs. For this correction bucket *single rates* are required and are obtained from list-mode processing (cf. “List-mode Data Processing”). **Note** that the discontinuities in the plots stem from the dead crystals acting as gaps between detector blocks

Sinogram profiles of all the transaxial components are shown in Fig. 8, including the geometric factors, crystal efficiencies, crystal interference, and detector dead-time. The geometric sinogram pattern (*a*) is repeated over all sinogram angles. The crystal efficiencies (*b*) are provided for each crystal separately, from which a unique normalisation factor is produced using two crystal efficiencies for a given sinogram bin. A sinogram pattern for transaxial crystal interference (*c*) is periodical due to the detector block repetition in the ring. A sinogram profile of dead-time factors only (*d*) is shown for three cases: (i) the first 15 seconds of amyloid acquisition where there are high single rates, (ii) the last 10 minutes and (iii) the overall average over the whole acquisition of 60 minutes. The greatest variability of the dead-time factors can be noticed in case (i) due to high singles rate variability. The gaps in the curves in Fig. 8 correspond to the LORs which have at least one dead crystal (acting as a gap between detector blocks).

### Axial normalisation factors

**Fig. 9:**
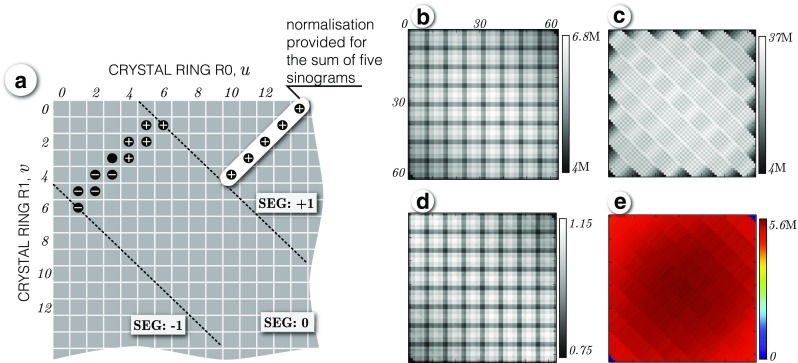
**Derivation of span-1 axial normalisation factors.** **a** Portion of Michelogram showing two native span-11 groups of five and six sinograms in segment ‘0’. For each group one normalisation axial factor is provided by the vendor (as shown for segment ‘+ 1’). **b** Michelogram of 24-hour cylindrical phantom acquisition in span-1. **c** Michelogram of the same phantom acquisition as in (b), but in span-11. **d** Michelogram of axial efficiencies derived using the phantom data and the vendor span-11 axial efficiencies according to Eq. (1). **e** Axially normalised phantom acquisition

The axial geometric component is captured within the general axial effects component, provided in the normalisation file for axial compression of span-11, whose organisation is demonstrated in the Michelogram space in Fig. 9a. Such axial compression makes image reconstruction faster and less memory demanding at the cost of some accuracy. Figures 9b and c show the Michelogram for the statistically rich prompt data of the ^68^Ge cylindrical phantom represented in span-1 and span-11, respectively. Figure 9d shows the derived span-1 axial normalisation factors according to Eq. (1), whereas Fig. 9e shows the Michelogram of the normalised emission data, where the patterns of slight imperfections can be noticed for the provided span-11 axial normalisation factors. To achieve best accuracy, all modelling (i.e., ray tracing) is always performed without any axial compression, that is in span-1, and hence using span-11 does not speed up computations, but instead it reduces data transfer times through lower memory usage.

**Fig. 10:**
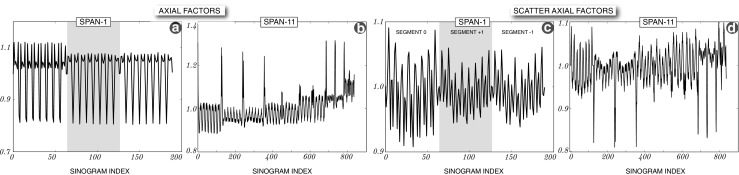
**Axial normalisation factors for span-1 and span-11**. **a**: Span-1 axial factors for the true events, derived from a long phantom acquisition and the provided span-11 axial factors; shown only for the first 190 sinogram planes (which constitute the first three segments of 64 + 2×63 sinogram planes) out of the total of 4084 sinograms. **b**: Span-11 geometric axial factors provided with the component normalisation file. **c**: Scatter specific span-1 axial factors derived from a high statistics phantom acquisition (only the first 190 sinogram plane factors are shown). **d**: Scatter span-11 axial factors, also derived from a phantom acquisition

The axial factors for span-11 (provided by the vendor) and the derived factors for span-1 are shown in Fig. 10 for true and scatter events. The span-1 factors for true and scatter events as derived in “Axial Normalisation Factors” (Fig. 9) are shown only for the first three segments, i.e., for 190 out of 4084 sinograms. There are 11 segments for span-11, while for span-1 there are 121 segments, cupped by the maximum ring difference of 60. For reduced axial FOV imaging, using a subset of detector rings, span-1 normalisation factors are used. Note that the sensitivity of the restricted ring system will be reduced compared to the full ring set, as fewer LORs will sample the image space. Consequently, the reconstructed images will exhibit higher noise levels compared to the full system. Alternatively, to prevent the reduction in sensitivity while achieving fast calculations, it is possible to compress span-1 data into single-slice rebinned (SSR) data and perform fast calculations on the compressed data. This, however, will reduce the accuracy and resolution of the system, while the proposed customisation maintains the resolution and accuracy, albeit at the cost of sensitivity and higher noise levels (the noise can be reduced by increasing the duration of acquisition in some cases).

The scatter axial factors are derived by reducing the span-1 scatter factors to span-11. Note that the scatter scaling in routine imaging not only appropriately scales the estimated scatter to the real data, but also accounts for the axial effects and outside FOV scatter specific for each scan, while the derived scatter normalisation accounts for the high frequency axial effects of detector blocks.

### Generation of the *μ*-map

**Fig. 11:**
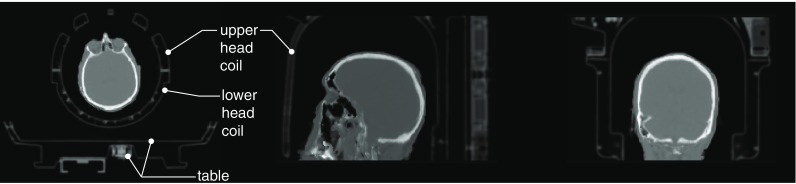
**Full composite** ***μ*** **-map of the patient and hardware.** Shown are transaxial, sagittal and coronal views, respectively

The accurate quantification is mostly dependent on the quality of the *μ*-map. Figure 11 presents the composite *μ*-map including: (i) the patient table together with the attached (ii) upper and lower head and neck coils, which were resampled to the PET FOV and image resolution; (iii) the high accuracy pCT-based *μ*-map of the head and neck for the amyloid brain scan. To account for head motion between time frames, the synthesised subject *μ*-map is coregistered to each PET time frame using reconstructed images without attenuation correction for better delineation of the head and more robust coregistration. Despite the high accuracy of the pCT-based *μ*-map, the coregistration of the *μ*-map to the PET image may introduce additional uncertainty caused by the limited precision of coregistration.

### Noise reduction of the estimated random events

**Fig. 12:**
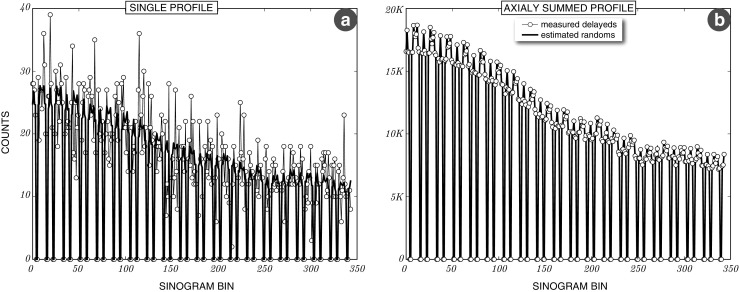
**Sinogram profiles of estimated randoms and measured delayed events**. **a**: Single sinogram profile for the measured delayeds and estimated random events of a 10 minute brain amyloid scan. **b**: The same profile but with all sinograms summed axially to obtain better statistics and demonstrate a good agreement of the measured delayeds and the estimated random events

The noise reduction of estimated randoms events allows for better quantitative precision. The extent to which the noise is reduced can be seen in a single span-11 sinogram profile for a 10 minute brain amyloid scan (50-60 minutes) shown in Fig. 12a. To check if the estimation is biased, all the direct and oblique sinograms (837 in total) were summed and the same sinogram profile location is shown in Fig. 12b. This demonstrates an unbiased random events estimation supported by the excellent agreement with the measured (summed for high statistics) counts of delayed coincidences in each sinogram bin.

### Voxel-based scatter model validation

**Fig. 13:**
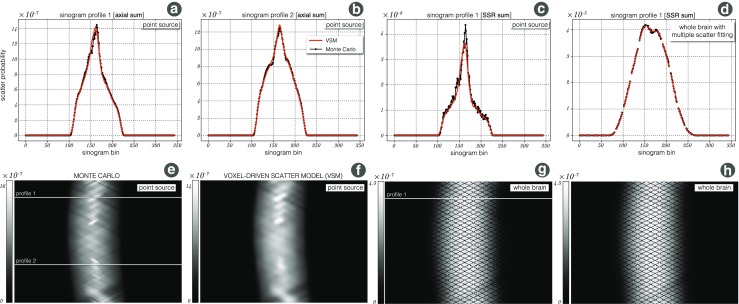
**Scatter validation using Monte Carlo (MC) SimSET simulations:** **a** Single scatter response to a point source *E* (see Fig. 5b) as estimated through the proposed VSM model (red) and the MC (dotted black) for sinogram *profile 1* as marked in (e); the sinograms were summed axially for good MC statistics. **b** The same as (a) but for sinogram *profile 2*. **c** The same sinogram *profile 1* as in (a) but the sinograms are single-slice rebinned (SSR). **d** Scaling of VSM estimate to multiple scatter MC sinogram for SSR sinogram *profile 1*. $\blacklozenge $ **e** The sum of all MC simulated single scatter sinograms with marked *profiles 1* and *2* for reference; **f** the corresponding VSM estimated scatter sinogram. **g** An SSR sinogram of the full brain MC simulation with marked *profile 1* for reference; **h** the corresponding full brain VSM estimated scatter sinogram (SSR summed)

The performance of the proposed fully 3D scatter model relative to the Monte Carlo (MC) simulation is presented in Fig. 13. The MC simulated single scatter sinograms were either summed axially to form one scatter sinogram with very good statistics or single-slice rebinned (SSR) to maintain some information of the axial scatter distribution at the cost of statistics. Figure 13a shows the agreement of the proposed VSM model (red) with the MC (black) for sinogram *profile 1* as marked in the axially summed MC scatter sinogram in Fig. 13e. Figure 13b shows the same but for sinogram *profile 2*. The corresponding axially summed VSM scatter sinogram is shown in Fig. 13f. The comparison with SSR scatter sinograms for sinogram *profile 1* is shown in Fig. 13c (fewer statistics in the MC sinograms with more axial specificity). Sinogram *profile 1* for the whole brain scan simulation is show in Fig. 13d as marked in the MC SSR sinogram in Fig. 13g. The corresponding VSM sinogram is shown in Fig. 13h. The VSM sinogram was fitted to the MC simulated multiple scatter in Fig. 13d. The presented figures demonstrate that the proposed scatter modelling can recover the scatter response with absolute probabilistic quantification for each emission voxel separately.

The trade-off between the accuracy of the model and the computational time is controlled by the resolution of the input *μ*-map and the current estimate of the emission image. In current settings for amyloid imaging, the input emission image is down-sampled by a factor of 3, resulting in a ∼ 6 mm emission voxel size, whereas for higher accuracy attenuation path calculations, the *μ*-map is down-sampled by a factor of two, resulting in a ∼ 4 mm voxel size. These settings result in the GPU computational time of 16 seconds per one iteration of the scatter forward modelling using the Tesla K20 graphics card. The scatter distribution as a response to a point source, contains high frequencies, mainly due to the sharp edges of the attenuating table and head coil (Fig. 13e and f). Despite the fact that the proposed scatter model uses only a limited set of transaxial and axial detectors, the high frequencies are well recovered at the point source response level. In practical settings, most scans consist of multiple point sources for which the high frequencies will likely disappear in the global scatter sinogram as can be seen in Fig. 13g and h. However, this will depend on the spatial distribution of the radiotracer. Furthermore, as shown in Fig. 13d, the proposed single scatter model can approximate multiple scatter by simple scaling to the scatter prompt data. This proves that for brain imaging single scatter modelling is sufficient even in the presence of the head coil.

### Uniform and Artefact Free Image Reconstruction

**Fig. 14:**
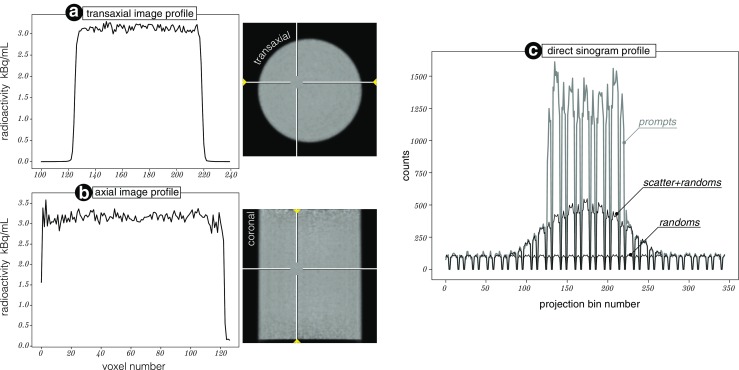
**Quantitative validation using the 20 cm** ^**68**^ **Ge cylindrical phantom:** **a** Transaxial image profile with its position marked by arrows in the image on the right. **b** Axial image profile with marked position in the coronal image on the right. **c** Direct sinogram profile corresponding to the transaxial image plane (*a*). The sinogram profile contains prompt data and the estimated scatter and randoms events with its agreement in the scatter-and-randoms-only regions. Note the detector gaps in the profile

Apart from the individual validations of attenuation, scatter and randoms, the software components are validated as a whole package with the long phantom acquisition. Figure 14 shows the transaxial (*a*) and coronal (*b*) image profiles of the reconstructed images shown on the right of the profiles. Note, that with this reconstruction it is possible to recover the global quantification of radioactivity per tissue volume [Bq/mL]. Furthermore, it was possible to obtain artefact free reconstructions indicating good performance of the normalisation component. The images and the profiles in Fig. 14a and b demonstrate good transaxial and axial uniformity, confirming accurate scatter and attenuation correction. In case of scatter inaccuracies, the images and profiles would most likely show a bump in the centre (scatter underestimation) or a dip (scatter overestimation). In the case of attenuation correction, note that the *μ*-map is not measured in the PET image space and hence has to be aligned precisely as otherwise even 0.5 mm will make visible non-uniformities in the image (visible at high count levels). Note the greater noise at the ends of axial FOV in Fig. 14b due to lower scanner sensitivity at those ends. In addition, the accuracy of the scatter and randoms estimates are shown in the projection space for one sinogram profile shown in Fig. 14c. The accuracy of the scatter estimate can be observed in the scatter-and-randoms-only regions, at both ends of the true component peak; whereas the fit of the estimated randoms can be seen at the far ends of the profile.

The absolute quantification in Bq/mL was achieved with a uniform cylindrical phantom scan with an independently and accurately measured sample from the phantom to obtain a single scaling factor, while ensuring high accuracy corrections for photon attenuation, scatter, detector normalization and dead time (Bailey et al. 1991). Although, such calibration is needed for some studies, it may not be needed for other studies, which use reference tissue regions for quantification (e.g., static standardised uptake value ratio [SUVR] or dynamic simplified reference tissue model estimates) (Meikle and Badawi 2005). In addition, the projector, together with the scatter model, can be used for simulating realistic PET projection data with different noise levels for any given digital phantom. Such comprehensively simulated data can then be reconstructed using *NiftyPET* in the research of optimal neuroimaging methods.

### Brain Imaging with Partial Volume Correction and Uncertainty Estimation

The presented methodology allows for a critical evaluation of the impact of PET count statistics on every part of the image reconstruction and analysis chain, including attenuation, scatter and randoms corrections, image registration (between MR and PET spaces), image interpolation (when resampling images due to registration and PVC), image segmentation/parcellation and PVC. This was achieved by resampling the list-mode data and generating 50 replications of the dataset, followed by repeating the image reconstruction and analysis chain in exactly the same way 50 times. This generated the distributions (uncertainties) of the estimated statistics of amyloid deposition at voxel and regional levels.

**Fig. 15:**
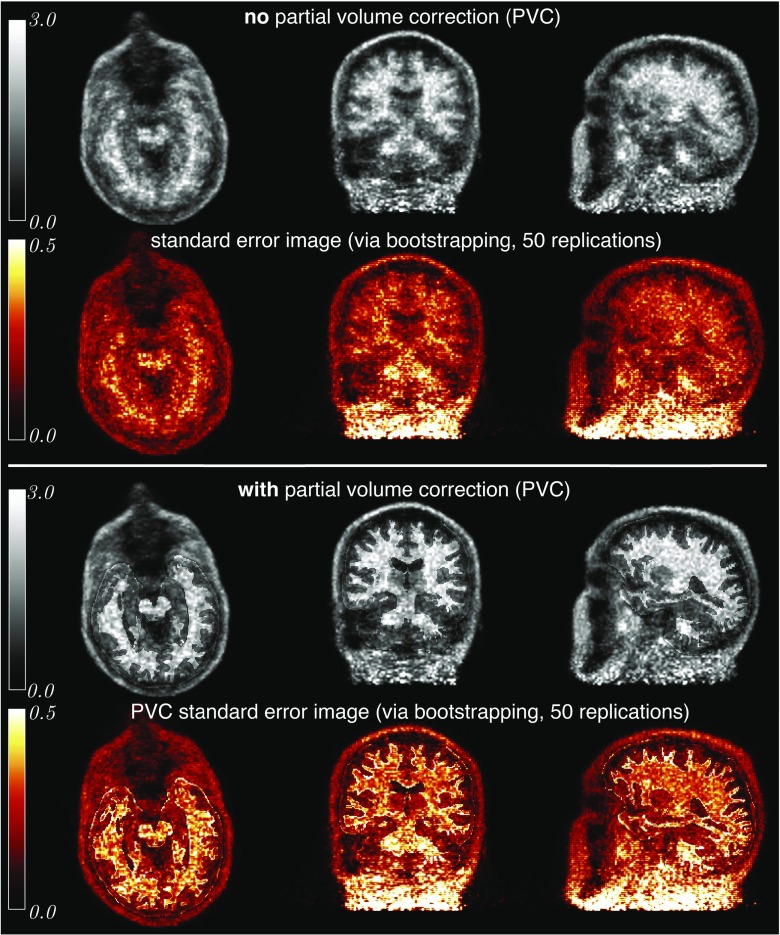
**Voxel-wise uncertainty estimation for PVC and non-PVC reconstructed images:** **Top two rows:** Four iterations of OSEM with 14 subsets (grey-scale) and the corresponding standard error image (copper-scale) after 50 bootstrap replications. **Bottom two rows:** The same as above but with added post-reconstruction PVC correction using the iterative Yang method (Erlandsson et al. 2012)

Figure 15 shows the results of the reconstructed images, normalised to the average of grey matter cerebellum, without (top row) and with the PVC (third row), producing SUVr images. The T1w image, acquired simultaneously with the source PET image on the Biograph mMR, was first parcellated with the specific regions of interest for amyloid imaging. Since the scanned participant was amyloid negative, most of the tracer uptake was in the white matter. The most conspicuous effect of the PVC is the improved delineation of the WM region, while the information about the tracer distribution within the region is preserved. This PVC correction is helpful in eliminating false positive amyloid measurements due to the spilling of WM radioactivity into GM regions.

Using the bootstrapping within the processing chain it was possible to generate standard error images (in copper colour scale) for both cases—with and without the PVC. Note, that despite the greater quantitative accuracy, the PVC introduces additional variability which can be explained by: (i) increased signal in the cortical regions from surrounding regions and thus increasing the variance, as well as (ii) the image registration which is slightly different for the various noise realisations of the PET data, consequently leading to the spatial variance of the brain parcellation. After normalising the standard error images by the corresponding mean images, it was observed that such normalised images for the cases with and without PVC are almost identical apart from the boundaries of the predefined ROIs where the variability is higher, likely due to the imprecision of MR-PET coregistration.

The voxel uncertainties, which can be quantified within *NiftyPET*, are greater at the end of the axial FOV due to the lower scanner sensitivity at this location. The presented voxel-level uncertainties can be reduced by considering larger regions instead of voxels.

With this capability of uncertainty estimation, it is possible to ascertain the impact of limited counting statistics in PET on all the different stages of image reconstruction and analysis, i.e., the image registration being inadvertently affected by the PET image noise, which consequently has an effect on attenuation and scatter corrections, partial volume correction and regional quantitative analysis. Furthermore, the counting statistics will have a direct impact on the scatter estimates and its scaling to the prompt data especially for short and noisy dynamic time frames. The same applies to the estimates of random events based on the measured delayed coincidences which are resampled the same way as the prompt events. All these aspects of error propagation through all of the image generation stages with their intricate dependencies are accounted for in the presented infrastructure using the efficient list-mode bootstrap resampling (cf. Markiewicz et al. 2016a). This may be useful in estimating the magnitude of errors in the measurement of FDG uptake in tumours (Kinahan and Fletcher 2010) or measuring the change in amyloid deposition in longitudinal studies of neurodegeneration (Landau et al. 2015); however such analyses go beyond the scope of this paper and constitute our future work. Currently, the software package is being further developed to include a richer library of reconstruction methods (Ehrhardt et al. 2016).

### Execution Timings

The performance was evaluated on three GPU cards. Two of the cards (NVidia’s Tesla K20 and TITAN Xp) were hosted separately on a Dell workstation (Precision T7600; 6-core Intel Xeon CPU E5-2630 @ 2.3 GHz; RAM: 64 GB @1333 MHz) and the other GPU (GeForce GTX 1080) was hosted in a Dell Alienware 17 R4 laptop (8-core Intel Core i7-7820HK CPU @ 2.90 GHz; RAM: 16 GB DDR4 @ 2667 MHz). The computational times were decomposed into four main stages and presented for the processing chain starting with the generation of the *μ*-map and finishing with a PVC image (see Table 1). Note that the laptop is newer than the Dell workstation, having a more efficient processor and faster memory and hence the execution times tend to be faster (transfers between CPU and GPU memory are faster). Scatter interpolation is the biggest bottleneck due to a CPU Python routine (not yet implemented on the GPU).

**Table 1:** Execution timings in seconds

	Host/Device		
Processing stage	Workstation/Tesla K20	Workstation/TITAN Xp	Laptop/GTX 1080
*μ*-map generation^∗^	78.6	72.2	65.83
LM processing ^⋆^	9.1	7.6	7.3
Image reconstruction^‡^	217.7	258.0	188.3
Scatter modelling	47.5	37.1	37.5
Scatter interpolation	190.4	193.7	122.87
PVC^†^	70.4	77.2	67.6

^∗^ Includes resampling of the UTE-based object and CT-based hardware *μ*-maps

^⋆^ Includes histogramming, bucket singles processing and motion detection.

^‡^ OSEM with 14 subsets and 4 iterations. Scatter correction is performed within the reconstruction.

^†^ Includes PET image upsampling, trimming and PET-MR image coregistration.

### Current and Future Developments

The *NiftyPET* package at this stage is available for Linux (e.g., Ubuntu, CentOS) and Windows systems. The package requires CUDA toolkit from NVIDIA (the latest version is available at https://developer.nvidia.com/cuda-downloads) and Python 2.7 (preferably Anaconda from Continuum Analytics, https://www.continuum.io/downloads). The GPU routines require a GPU card with the compute capability of at least 3.5 (NVIDIA 2017b).

The package is currently being extended to support all the PET/MR scanners (with and without TOF) deployed in the United Kingdom within the Dementias Platform UK network (DPUK), for harmonised image reconstruction and analysis in multi-centre clinical trials. *NiftyPET* is actively being developed to support TOF-PET, with already added support for TOF scatter estimation (Markiewicz et al. 2016b), which needs further validation. Also, a separate module for accurate and robust motion detection is under development to expand upon previous work on the Microsoft Kinect (Noonan et al. 2015) for frame-by-frame and direct list-mode reconstruction.

At this stage, *NiftyPET* supports only Siemens mMR PET/MR scanners, nevertheless, it can readily be adapted to other Siemens scanners as they share the same list-mode data format and similar technological solutions. For full quantification with *NiftyPET*, it is recommended that each scanner is calibrated against a laboratory standard, e.g., using a uniform phantom scan and relating it to a well-counter (Bailey et al. 1991). The support for GE scanners (including the GE Signa PET/MR scanner) is actively being developed. The support of Philips scanners can be added when all the necessary scanner’s specifications are available. While not covered in this paper, *NiftyPET* supports dynamic LM processing (Markiewicz et al. 2016a) and reconstruction, and is under further development to incorporate advanced kinetic modelling based on either independent time-frame reconstruction (a fast option) or joint estimation of kinetic parameters with head motion (slower and computationally demanding, see Jiao et al. (2017)). Although developed primarily for brain imaging and analysis using PET/MR scanners, *NiftyPET* can be used for whole body imaging, including PET/CT scanners.

Currently, the *NiftyPET* package only allows for EM (or OSEM) reconstruction. However, as the software package is very modular and python interfaces are available, it can be used in conjunction with other packages (e.g., ODL; https://github.com/odlgroup/odl) to reconstruct from PET data with any reconstruction model, such as maximum a-posteriori reconstruction (Tsai et al. 2015; Comtat et al. 2002; Ehrhardt et al. 2016) or Bregman iterations (Müller et al. 2011; Benning et al. 2013; Osher et al. 2005) with any kind of prior (Burger and Osher 2013; Ehrhardt et al. 2016; Liao and Qi 2007) or algorithm (Chambolle and Pock 2016; Chambolle et al. 2017; Tsai et al. 2015; Comtat et al. 2002).

## Conclusions

We have presented an open source Python package *NiftyPET* for image reconstruction and analysis with high quantitative accuracy and precision as well as with uncertainty estimation, while facilitating high-throughput parallel processing using GPU computing. We put a particular emphasis on the software’s high quantitative accuracy for brain imaging using the PET/MR scanners—in particular, the attenuation correction using accurate *μ*-maps, fully 3D scatter modelling with high resolution ray tracing, randoms estimation, and fast image convolutions for PVC. The rapid list-mode data processing enables generation of independent bootstrap realisations, which in turn allow fast uncertainty estimation of any image statistic. We have also extended the Siemens default span-11 image reconstruction to span-1 (no axial compression), which is particularly useful when reducing the large axial FOV of the PET/MR scanner to a narrower FOV and thus enabling much faster reconstructions with real data—a unique feature which is useful for validating new reconstruction or analysis methods (e.g., kinetic analysis) through multiple noise realisations (rapidly generated by the bootstrap).

## Information Sharing Statement

All the presented software is open-source and available at https://github.com/pjmark/NiftyPET (RRID:SCR_015873), which contains a wiki on installation and usage. The PET/ MR data used here will also be available for download, which, together with the provided Jupyter Notebook files, will enable independent recreation of the presented figures in a straightforward manner.
